# Development and Characterization of a Microemulsion Containing a Cannabidiol Oil and a Hydrophilic Extract from *Sambucus ebulus* for Topical Administration

**DOI:** 10.3390/pharmaceutics16060705

**Published:** 2024-05-24

**Authors:** Teresa Areses-Huete, Damian Cordoba-Diaz, Ana Isabel Torres-Suárez, Manuel Cordoba-Diaz

**Affiliations:** 1 Department of Pharmaceutics and Food Technology, Faculty of Pharmacy, Complutense University of Madrid, E-28040 Madrid, Spain; marese01@ucm.es (T.A.-H.); damianco@ucm.es (D.C.-D.); galaaaa@ucm.es (A.I.T.-S.); 2 University Institute of Industrial Pharmacy (IUFI), Complutense University of Madrid, E-28040 Madrid, Spain

**Keywords:** cannabidiol, microemulsion, phytocannabinoids, rheological properties, diffusion studies, vegetal extract

## Abstract

Cannabidiol (CBD) is a safe and non-psychotropic phytocannabinoid with a wide range of potential therapeutic anti-inflamatory and antioxidant activities. Due to its lipophilicity, it is normally available dissolved in oily phases. The main aim of this work was to develop and characterize a new formulation of a microemulsion with potential anti-inflammatory and antioxidant activity for the topical treatment of inflammatory skin disorders. The microemulsion system was composed of a 20% CBD oil, which served as the hydrophobic phase; Labrasol/Plurol Oleique (1:1), which served as surfactant and cosurfactant (S/CoS), respectively; and an aqueous vegetal extract obtained from *Sambucus ebulus* L. (*S. ebulus*) ripe fruits, which has potential anti-oxidant and anti-inflammatory activity and which served as the aqueous phase. A pseudo-ternary phase diagram was generated, leading to the selection of an optimal proportion of 62% (S/CoS), 27% CBD oil and 11% water and, after its reproducibility was tested, the aqueous phases were replaced by the vegetal hydrophilic extract. The defined systems were characterized in terms of conductivity, droplet size (by laser scattering), compatibility of components (by differential scanning calorimetry) and rheological properties (using a rotational rheometer). The designed microemulsion showed good stability and slight pseudo-plastic behavior. The release properties of CBD from the oil phase and caffeic acid from the aqueous phase of the microemulsion were studied via in vitro diffusion experiments using flow-through diffusion cells and were compared to those of a CBD oil and a microemulsion containing only CBD as an active substance. It was found that the inclusion of the original oil in microemulsions did not result in a significant modification of the release of CBD, suggesting the possibility of including hydrophilic active compounds in the formulation and establishing an interesting strategy for the development of future formulations.

## 1. Introduction

Cannabinoids are a group of compounds that can exert different homeostatic and pharmacological effects through the so-called endocannabinoid system (ECS), a network of molecular signaling made up of a group of signaling molecules known as endocannabinoids, a group of cannabinoid receptors (CB1, mostly located in the central nervous system, and CB2, predominantly distributed in peripheral and immune cells) and some enzymes that module the synthesis and breakdown of the signaling molecules and their transporters [1,2]. Many authors have described the importance of cannabinoids for the potential treatment of different inflammatory disorders in which the ECS is involved at different levels [3,4]. 

The importance of the ECS in the skin is now well known due to the presence of CB1 and CB2 receptors identified in different layers of the skin, evidence which supports the potential therapeutic uses of dermal and transdermal administration of cannabinoids for the treatment of different chronic or occasional inflammatory skin disorders such as psoriasis, pruritus, urticaria and atopic dermatitis [5,6,7,8,9,10].

Cannabidiol (CBD) is the most abundant non-psychotropic cannabinoid found in the cannabis plant. Its high safety profile shows great potential and has a wide range of therapeutic uses that have been increasingly described since 1963, when its structure was first reported [11,12]. Concerning the mechanism of action of CBD, it is known that its anti-inflammatory effect can be ECS-receptor-dependent or non-ECS-receptor-dependent. In the first mechanism, CBD exerts its activity via cannabinoid receptor CB2 [11]. 

Many authors have also described a significant non-ECS-receptor-dependent activity for CBD. Carrier et al. [13] suggested that CBD has the ability to enhance adenosine activity (via A2A receptors), provoking a decrease in inflammatory activity and downregulation of over-reactive immune cells. Bisogno et al. [14] described the agonist effect on TRPV1 (transient potential vanilloid receptor type 1) due to the analogy of CBD with capsaicin, which allows it to exert similar effects (without noxious effects). Russo et al. [15] consider that CBD can act as an agonist to the serotonin receptor (5-HT1A), as is reflected in consequent activity, and they also described CBD activity as an antagonist of GPR55 receptors, which leads to a decrease in IL-12 and TNF-α production [16,17]. It has been also found that CBD can act through ion channels as an agonist and inhibit enzymes such as phospholipase A2, cyclooxygenase 1 and 2 and fatty acid amide hydrolase (FAAH), which is responsible for the diminution of arachidonic acid levels [18].

All of these actions provide strong anti-inflammatory and antioxidant activity, as well as the appropriate immunological response activity, which is reflected in a wide variety of potential therapeutic uses [19]. Hence, the most widely reported effects of CBD in the literature are anti-inflammatory and antioxidant [11,12,16,17,20,21,22], but there exists evidence of other effects related to the treatment of skin disorders [1,16,20,21,23], arthritis [11,12,16,17,22,24], diabetes [11,16], neurodegenerative diseases [2,11,16,22,25] and epilepsy [11,16,22,25]. CBD has also been reported as a very promising drug for the treatment of anxiety and depression and for sleep regulation [11,16,19,20,21,22,24,25], as well as for the treatment of chronic pain [17,26] and as a complementary treatment for different types of cancer [11,19,22,27].

Chemically, CBD is a terpene–phenol exhibiting affinity for oil phases due to its poor solubility in water. The high lipophilicity of CBD conditions its pharmacokinetic properties. Orally administered CBD shows very low bioavailability (around 6%) because the hepatic first-pass metabolism is very high (over 95%) [22,28]. There have also been reports of variable pharmacokinetic profiles and complications in the dosage forms, leading to unpredictable side effects and multiple interactions with other substances because CBD is metabolized into a competitive inhibitor of CYP-450 enzymes [19,24]. 

The physicochemical characteristics of CBD condition its therapeutic efficacy by different routes of administration, which has made it necessary to find strategies to protect or/and encapsulate this active substance with the aim of improving its pharmacological activity and minimizing side effects. Many attempts have been made to design an optimal CBD formulation for topical administration due to its lipophilicity and the inherent potential of therapeutic use of phytocannabinoids for the treatment of skin disorders [29,30]. A CBD solution using propylene glycol as a vehicle showed interesting transdermal absorption in vitro, with the formulation including some essential oils and oleic acid as penetration enhancers [31]. Dimethyl sulfoxide has also been proposed as a vehicle for CBD in in vitro experiments to confirm its efficacy in the treatment of allergic contact dermatitis [32]. This solvent can act as a penetration enhancer and solubilizer but cannot be used as a main vehicle in a topical formulation due to its poor organoleptic and potentially irritant properties. Other options like the use of CBD in a shampoo have demonstrated its efficacy for the treatment of scalp psoriasis and dermatitis [33]. Some cosmeceuticals formulated in cream or gel have also been described as effective for the topical treatment of pruritus [34]. Zielińska and co-workers [21] recently published an interesting review on the latest examples of formulation of CBD in lipid systems for topical administration, mainly in the form of emulsions, and included additional technologies like lipid nanoparticles. Many examples of commercially available CBD oils registered as cosmeceuticals or medical devices exist as well. In the light of the availability of these products, some studies have been conducted in the last few years to demonstrate their therapeutic efficacy in topical treatments [35,36]. Although mere oils could constitute an ideal vehicle for CBD due to its lipophilicity, this simple formulation does not permit the concomitant inclusion of other hydrophilic molecules with potential antioxidant, anti-inflammatory or antibiotic activities, etc., let alone aqueous extracts from different plants. In those cases, the use of emulsions, liposomes and any other system that permits the inclusion of oils and aqueous phases is required.

In our study, the formulation of a CBD oil concomitant with the formulation of an aqueous extract of a plant showing antioxidant and anti-inflammatory effects to enhance the activity of CBD was proposed. *Sambucus ebulus* was selected due to its reported inherent properties. *Sambucus ebulus* sp. is one of the many species of the *Sambucus* genre. Also known as “dwarf elder”, it grows all over Europe and South Asia and is found in many territories of America. This herb grows approximately 1.5–2 m high in humid and luminous areas (near rivers) with large and extensive groups of underground rhizomes [37,38]. The most commonly reported pharmacological properties are the potential antioxidant and anti-inflammatory effects of the aerial parts: mainly the fruits, leaves and flowers [39,40,41,42], although some authors also describe promising anti-inflammatory activity in its rhizomes [43,44]. Recently published studies [45] confirm the anti-inflammatory and antioxidant activities of different botanical parts of *S. ebulus*. There also exists considerable evidence that several formulations using different extracts from *S. ebulus* show a clear anti-inflammatory effect [46]. For example, Saberian et al. [47] showed the efficacy of a gel containing an extract from *S. ebulus* for the treatment of pruritus and also demonstrated its wound-healing capacity, which was due to the anti-inflammatory properties. Different studies published in the last few years describe the analgesic and anti-inflammatory effect of a 5% topical ointment of *S. ebulus* fruit extract, showing favorable skin-healing effects in the treatment of cutaneous leishmaniasis [48]. The anti-inflammatory effects of these extracts have been also compared to those of different drugs like diclofenac [37,44] or sodium salicylate [43]. Recent studies [49] show some promising results concerning the effects of the oral administration of *S. ebulus* fruit extract on the regulation of the pro-inflammatory response, which resulted in a positive effect on the immuno-modulation mechanisms of volunteers.

Anti-inflammatory activity is not the only reported property of *Sambucus ebulus*. Jabbari et al. in 2017 [37] described its potential for use in the treatment of metabolic disorders, as well as its potential as an antidepressant, its potential neuroprotective, antioxidant, analgesic and wound-healing activities, its potential positive effects on knee osteoarthritis and its potential antimicrobial and antigiardial activities, among others.

Different phytochemical compounds from *S. ebulus* have been reported to be responsible for the previously-described therapeutic effects, including steroids, tannins, flavonoids and phenolic acids. Caffeic acid (CAF) is a hydroxycinnamic-derived acid thoroughly described by several authors. This phenolic acid is one of the compounds that can be found naturally in the extracts obtained from *Sambucus ebulus*, specially in extracts obtained from mature fruits, and due to its antioxidant and anti-inflammatory activity and its hydrophilic characteristics [50,51], it is an interesting compound in the context of our study. CAF was selected as a model hydro-soluble and antioxidant compound through which to characterize the obtained extract and the microemulsion due to its abundance in the extracts and also because CAF can be easily analyzed by fluorescence, as previously described in the literature [52], using the same chromatographic conditions selected for the analysis of CBD. Apart from its pharmacological relevance, CAF constitutes a good hydrophilic model compound through which to study the release of substances from the aqueous phase of a multi-phasic system like an emulsion or a microemulsion.

The strategy of obtaining encapsulated oils has been proposed by many authors for the production of pharmaceutical formulations intended for the treatment of a variety of health problems and diseases because such formulations provide more protection against oxidation when compared to unencapsulated oils. Microemulsions have been shown to be effective vehicles for the solubilization of different drugs, as well as a protective medium against degradation by light exposure, oxygen, etc., preventing hydrolysis and oxidation reactions. A prolonged release of the drug can increase bioavailability across different administration routes and prevent irritative or toxic effects of the active ingredient [21,53,54]. 

Microemulsions are defined as a transparent and isotropic dispersion of an aqueous and an oil phase structured as a bi-continuous unique phase or a discontinuous system at a nanometric scale (10–100 nm droplet size) stabilized by appropriate proportions of both phases in combination with surfactants and co-surfactants [55,56]. Unlike emulsions, such systems have the advantage of being thermodynamically stable and easier to prepare, since simple stirring to mix the components of the formulation will be enough to generate the formulation, providing the correct proportions of both phases and amphiphile components is obtained. Apart from its better stability and its simplicity of preparation, even at the industrial scale, microemulsions have important advantages, showing interesting rheological properties like low viscosity [57], the possibility of sterilization, and their capacity to solubilize both hydrophilic and lipophilic drugs [58,59]. Hence, microemulsions constitute a compelling strategy for the vehiculization of many different substances by different administration routes.

In our study, a preformulation study was performed using purified Milli^®^-Q water as a hydrophilic phase in order to accurately define the optimum composition of the microemulsion. Subsequently, water was replaced by an aqueous extract obtained from ripe fruits of *Sambucus ebulus* L. The final formulation was characterized using the same protocol described for the previous microemulsion to be sure that the physicochemical properties were not altered with the inclusion of the extract. The stability of the microemulsion system was also assessed in terms of physical and chemical properties under room-temperature storage conditions.

The main aim of this work is focused on the development and characterization of a new microemulsion formulation obtained from a CBD oil, including the determination of the components and the proportions needed to obtain a stable microemulsion, compatibility studies and release characteristics, with the intent of including the maximum proportion of CBD oil concomitantly with an aqueous *S. ebulus* extract to reinforce its potential synergic anti-inflammatory activity for the treatment of topical disorders, including psoriasis, etc. Such a formulation could expand the possibilities for new therapeutic perspectives. 

To our knowledge, the most similar approach to this strategy was published by Vanti and co-workers [60], who described a gel based on an O/A microemulsion loaded with 1% cannabidiol. The microemulsion developed in the present study would allow the future inclusion of different hydrophilic compounds together with CBD in a thermodynamically stable system without significantly modifying the rheological and release properties of the original oil. The idea of reinforcing the pharmacological activity of the CBD oil with hydrophilic compounds providing a potential synergic effect could result in important improvements in the therapeutic use of CBD for the treatment of different skin disorders.

## 2. Materials and Methods

### 2.1. Chemicals and Reagents

Labrasol^®^ (PEG-8 caprylic/capric glycerides) and Plurol-Oleique ^®^ CC497 (Polyglyceryl-3 oleate) were selected as the surfactant and co-surfactant additions to the microemulsion formulation and were kindly supplied by Gattefosé-Spain, Madrid, Spain.

Cannabidiol, 99.9% crystals, as well as Beemine CDB oil Forte+^®^ 20%, were kindly supplied by Beemine Laboratories (Madrid, Spain). The commercially available CBD oil contained CBD in solution (20% *w*:*w*) and an oil mixture of hemp (majority component) and sunflower seed oil as the vehicle.

Ripe *Sambucus ebulus* L. fruits were collected (40°10′15″ N 4°24′19″ W) when optimal ripening conditions were assured and taxonomically characterized at the MAF herbarium (assigned number MAF 176062), which is located at the Faculty of Pharmacy of the Complutense University of Madrid.

Fruits were cleaned, frozen and stored at −20.0 ± 0.5 °C until use. The extract was produced as follows: an accurately weighed amount of thawed fruits were digested in methanol for 24 h and filtered. The resulting methanolic solution was dried using a Buchi^®^ R-100 rotary evaporator and a Telstar LyoQuest^®^ freeze dryer (Terrassa, Barcelona, Spain) to ensure the total elimination of liquid solvents and stored at −20.0 ± 0.5 °C until use. The dry extract was redissolved in purified water before use. 

All reagents and chemicals were of analytical grade. Folin-Ciocalteu′s phenol reagent was purchased from Sigma-Aldrich, (Madrid, Spain). Methanol, acetonitrile and ethanol were purchased from Fischer Chemical Spain and were supplied as HPLC reagent-grade. Milli-Q^®^ water was used for all the experiments.

### 2.2. Quantification of Cannabidiol and Caffeic Acid by HPLC

A previously developed and validated RP-HPLC method was used according to the Q2 ICH guidelines for the simultaneous quantification of CBD and Caffeic Acid (CAF). Briefly, a JASCO modular high-performance liquid chromatograph (Jasco International Co., Ltd., Tokyo, Japan) equipped with a LG-2080-04 quaternary low-gradient unit, a PU-2080 pump, a DG-2080-54 degasser and an AS-2050-plus autosampler was used. The equipment had two detectors to allow it to collect an absorbance signal (UV-2070 plus UV/Vis detector) and fluorescence (FP-4025 Fluorescence detector) (Jasco International Co., Ltd., Tokyo, Japan). 

A chromatographic C18 column (Mediterranea^®^ Sea, 5 µm, 150 × 4.6 mm) (Teknokroma S. Coop., Madrid, Spain) thermostated at 40 °C in a Jasco column oven 2065 Plus was selected as the stationary phase. The mobile phase consisted of acetonitrile: methanol: acid–water solution (adjusted to pH 4.5 with glacial acetic acid) 30:52:18, pumped at a rate of 1.8 mL·min^−1^. The injection volume was fixed to 20 µL, and the run time for data collecting was set to 7 min. Detection was carried out spectrophotometrically for CBD at a wavelength of 228 nm, resulting in a repetitive peak at 4.8 min. The simultaneous analysis of CAF was carried out by spectrofluorimetry, selecting 262 and 426 nm for excitation and emission wavelengths, respectively. A repetitive peak for caffeic acid at 2.1 min was obtained.

For the developed method, linearity (R^2^ = 0.9999) was observed in the range of 11.2–500 μg/mL. Limit of detection (LOD) and limit of quantitation (LOQ) were estimated at 3.7 and 11.2 μg/mL, respectively, for CBD, and 5.9 and 18.1 µg/mL for CAF. Both values were calculated from the standard deviation of the linear response and the slope of the calibration line, as described in the ICH Q2(R2) Guidelines. The method was accurate and precise inter-day and intra-day (RSD of 3.35 and 3.61%, respectively), with accuracy values of 100.63% for CBD and 99.7% for CAF.

### 2.3. Design and Preparation of the Microemulsion System

The microemulsion was produced using the aqueous titration method, as described in previous papers [56]. Briefly, the 20% CBD oil constituting the oily phase was combined with different proportions of surfactant/co-surfactant (S/CoS) mixtures (Labrasol^®^ and Plurol-Oleique ^®^ CC497, respectively, 1:1 gravimetric ratio) under stirring at 30 °C for 10 min. The obtained mixtures were titrated with purified water under magnetic stirring at the same temperature, and the resulting microemulsion was then allowed to stabilize for 20 min. The composition of different mixtures obtained are shown in Table 1. A pseudo-ternary phase diagram was produced to determine the microemulsion area. When idoneal microemulsion area was determined, the experiment was repeated, replacing Milli-Q^®^ water with the aqueous fruit extract previously produced.

### 2.4. Physicochemical Characterization of Microemulsion

As previously specified, the main objective of this study focused on including an aqueous phase while preserving the maximum proportion of CBD oil. The aqueous phase would be used for the future incorporation of different hydrophilic compounds with potential therapeutic uses like antioxidant, anti-inflammatory and antibiotic effects, etc. into a microemulsion, while preserving as much as possible the original properties of the CDB oil in terms of rheological behavior, spreadability and drug release. Based on this premise, the microemulsion containing a proportion of S-CoS:O:W of (62/27/11)and a CBD load of 5.4% (*w*:*w*) was selected for further characterization studies.

#### 2.4.1. Conductivity

The measurement of electrical conductivity is widely described as an adequate method through which to study structural changes in biphasic systems like emulsions and microemulsions [57]. In our study, conductivity measurements were carried out as described in a previous paper [56]. Briefly, a METROHM^®^ 644-Conductimeter (Metrohm Hispania, Madrid, Spain) equipped with a CRISON^®^ conductivity cell was used under continuous stirring at a constant temperature of 30 °C while the titration of the microemulsion by different amounts of Milli-Q^®^ water was performed.

#### 2.4.2. Droplet-Size Distribution

A MICROTAC INC^®^ instrument (MBT, Madrid, Spain) equipped with a Zetatrac software 10.5.3 was used for the measurement of the mean droplet size and the size distribution by laser diffraction. All the measures were conducted in triplicate from three samples of microemulsion and emulsion, using purified water as a blank in both cases. Semi-logarithmic plots of droplet-size distribution were obtained for each formulation.

#### 2.4.3. Rheological Properties

A DVNext Brookfield cone/plate rotational rheometer equipped with a CP42 cone spindle was used to determine the apparent viscosity as shear rate increased and decreased sequentially (0–61.4–0 s^−1^; 255 s; 25 °C). Upward and downward curves corresponding to shear stress (SS) vs. shear rate (SR) and SR vs. viscosity (mPa·s) were collected using the internal Rheocalc software DVNXRVCJG 2.1.8.3-9 of the equipment. The power-law model of Ostwald–de Waele was used to analyze the possible structural changes in borg the original CBD oil and the CBD included in the microemulsion system as the oil phase [61,62]. Briefly, the SS (D·cm^−2^) vs. SR (s^−1^) rheograms were adjusted to the model defined by Equation (1), which is as follows:

(1) $$SS=K\cdot {SR}^{n}$$

where K is the consistency coefficient and n denotes the flow-behavior index or power-law index (1 in Newtonian fluids and lower than 1 in pseudoplastic systems). The shear properties of the microemulsion can also be characterized by the Herschel–Bulkeley model, according to Equation (2), as follows:

(2) $$SS-{SS}_{0}=K\cdot {SR}^{n}$$

where SS_0_ is the yield stress, defined as the minimum shear stress required for the formulation to initiate flow. 

The thixotropy was evaluated through hysteresis (area between the ascending and descending curves), where a greater area meant a greater thixotropic effect.

#### 2.4.4. Compatibility Studies

There exist several approaches through which to study the chemical compatibility of drug–excipients for the development of new formulations. One such is differential scanning calorimetry (DSC), a rapid and easy technique used in this field [63] that is very useful for the detection of potential incompatibilities in the early stages of developing a new product. In our study, the chemical compatibility of CBD with the other components of the formulations, 20% CBD oil and the designed microemulsion, was determined by DSC. 

DSC experiments were conducted on CBD crystals and fluid formulations (CBD oil and microemulsion) using a TA Instruments DSC-Q200 (Waters-TA Instruments, Barcelona, Spain). Quantities ranging from 3 to 10 mg, depending on the kind of sample, were placed in a closed aluminum crucible. The heating rate was 10 °C/min, with programming temperatures from 35 to 535 °C. All the experiments were conducted under nitrogen atmosphere at a flow rate of 50 mL·min^−1^.

#### 2.4.5. In Vitro Drug Release

The release characteristics of CBD from the developed microemulsion system were determined in vitro in comparison to those of 20% CBD oil using flow-through cells. Data were mathematically studied using a consecutive-reaction model simplified to a growth model, as described in a previous paper [64]. The release of caffeic acid from the microemulsion containing the *S. ebulus* extract as an aqueous phase was also studied using the same methodology. Briefly, the diffusion equipment (PermeGear^®^ ILC-07 automated system (Riegelsville, PA, USA) consisted of seven in-line flow-through diffusion cells made of Kel-F, in which the donor and receptor chambers and the diffusion membrane (SpectraPor^®^—LE dialysis Cut off 10,000 Da), were placed over a support with a hole of 1 cm in diameter (diffusional area, 0.785 cm^2^). Tygon tubing was used for all the inlet and outlet connections to prevent drug adsorption. The cells were thermostatized at 37 °C and placed in a cell warmer connected to a Haake -DC10^®^ circulating bath (Gebruder Haake, Karlsruhe, Germany). An Ismatec^®^ IPC-16 peristaltic pump (Ismatec, Zurich, Switzerland) was used to provide a constant flow rate of 1 mL·min-1 for the receptor medium (ethanol/water mixture 70:30 *v*:*v*). Samples were collected in glass receptor tubes from an Isco^®^ Retriever IV fraction collector (Isco, Lincoln, NE, USA). An Indexing Controller (also available from PermeGear) was used to independently program the duration of each shuttle’s time in the retriever so that 19 samples could be collected simultaneously from each cell. 

The evolution of the CBD and CAF concentrations that permeated to the receiver chamber (C_rec_) versus time (t) was studied by using the following equation, which was proposed by Harrison et al. for flow-through diffusion cells [65]:

(3) $${V_{rec}\cdot dC}_{rec}/dt=J\cdot A-F_{rec}\cdot C_{rec}$$

where V_rec_ is the volume of the receiver chamber; J is the apparent intrinsic flux of the drug through the membrane; A is the diffusional area and F_rec_ is the flow rate of the receptor fluid. The term dC_rec_/dt was easily estimated from the concentration-versus-time raw data. The permeability coefficient (Kp expressed in mg·h^−1^) was calculated from the J value divided by the concentration in the donor chamber at time zero according to the Fickian diffusion model.

The release kinetic constants of the lipophillic CBD and the hydropillic CAF from the formulation were calculated from the profiles of cumulative permeated amounts of drug vs. time using the following expression, which was derived from the mathematical model of the first-order consecutive reactions [66]:

(4) $$Q_{rec}=Q_{0}\left[1-\left(1+\frac{K_{1}}{K_{2}}\right)e^{-K_{1}t}\right]$$

where Q_rec_ is the cumulative amount of drug permeated to the receptor chamber at each time and Q_0_ is the initial amount of drug into the donor compartment. K_1_ (h^−1^) is the kinetic constant expressing the release of the drug from the formulation, and K_2_ (h^−1^) expresses the diffusion through the membrane to the receptor chamber. First-order kinetic behavior was assumed for comparison between the two formulations and was tested as a concentration-dependent release process. This assumption is based on the fact that a finite-dose diffusion system is obtained and the assumption that the amount of drug applied is sufficient to sustain a constant rate of absorption over the study duration and that a steady state is not maintained.

Analysis of data was carried out using the GCFIT program (SIMFIT package 6.0.24. W.G. Bardsley, University of Manchester). Best fit was achieved using growth/survival mathematical models based on the function A[1-exp(K·t)] for all experiments. Goodness of fit was determined by the analysis of residuals and Durbin–Watson test, as well as the Shapiro–Wilks and Akaike AIC stats. All data were expressed as mean ± confidence interval. A *p* value < 0.05 was considered to be statistically significant using the *t*-test between the two means for the unpaired data. Data analysis was conducted with SPSS software, version 14.0 (SPSS Science, Chicago, IL, USA). 

### 2.5. Antioxidant Activity and Total Phenolics of the Microemulsion-Extract

Total phenolics content (TPC) and total antioxidant capacity (TAC) of the microemulsion were determined by using Folin–Ciocalteau and CUPRAC (cupric reducing antioxidant capacity) methods, respectively, following the protocols described by Karadirek et al. (2016) and Flores (2017) [67,68] to assess the integrity of the antioxidant compounds after the extraction process with methanol and the subsequent inclusion of the extract in a microemulsion system.

Briefly, the TPC method is based on the reduction of phenolic compounds in the presence of the Folin–Ciocalteu reagent (Sigma-Aldrich Spain), provoking a colorimetric reaction that can be quantified spectrophotometrically at a wavelength of 760 nm. TPC results were expressed as equivalent micrograms of gallic acid. A calibration curve was previously established by analyzing different concentrations of gallic acid standard solutions as follows: 1.8 mL of Na_2_CO_3_ 7.5% *w*:*v* aqueous solution, 0.6 mL of Folin–Ciocalteu reagent, and different amounts of a 1.25 mM gallic acid solution were added to each glass tube, with the volume brought up to 4.5 mL with Milli-Q^®^ water. The standard solutions were incubated for 60 min in darkness at room temperature, and the absorbance was measured in the interval from 12.8 to 63.9 µg equivalents of gallic acid. Linearity, repeatability and accuracy were validated within the range of gallic acid concentrations measured. Extract and microemulsion samples were prepared in the same way as the standard solutions at different concentrations to determine the average TPC as µg equivalents of gallic acid per µg of dry extract.

TAC was determined using CUPRAC reagent (neocuproine—Merk, Madrid, Spain), using a methodology similar to that previously described for the TPC method: 1 mL of CuCl_2_ 10 mM solution, 1 mL of 0.5 mM neocuproine solution, 1 mL of 1 M ammonium acetate and different amounts of 1 mM gallic acid solution were added to each glass tube, with the volume brought up to 4 mL with Milli-Q^®^ water. Samples were incubated and quantified spectrophotometrically at a wavelength of 450 nm. Equivalent amounts of gallic acid from 3.4 to 17 µg were found to have good linearity, accuracy and repeatability. Extract and microemulsion samples were prepared in the same way as the standard solutions at different concentrations to determine the average TAC as µg equivalents of gallic acid per µg of dry extract.

## 3. Results and Discussion

### 3.1. Preparation of the Pseudo-Ternary Phase Diagram and Design of the Microemulsion System

For the design of the formulation as a microemulsion system, Labrasol^®^ (PEG-8 caprylic/capric glycerides) and Plurol-Oleique^®^ CC497 (Polyglyceryl-3 oleate) were selected as the surfactant and co-surfactant, respectively, due to their biocompatibility and to their effectiveness for the formation of a microemulsion in a 1:1 gravimetric ratio, as determined in previous studies [56]. These excipients are also widely used in formulations intended for topical administration in cosmetic or pharmaceutical use.

**Figure 1:**
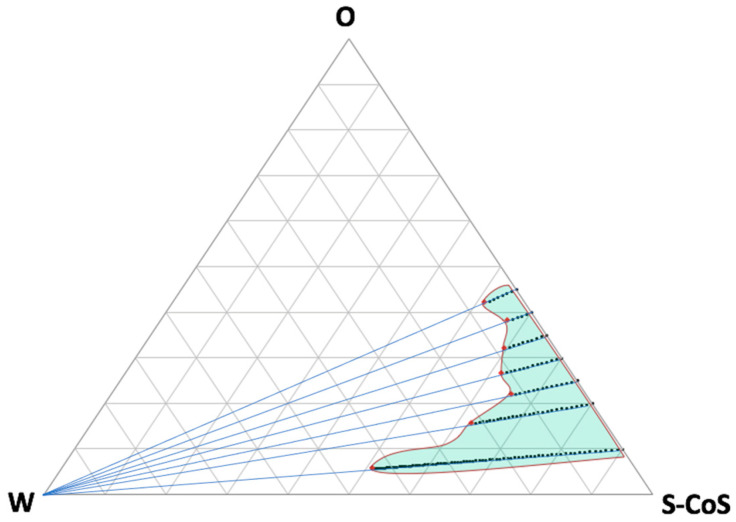
Pseudo-ternary phase diagram showing all the tested titration lines up to the limits of the microemulsion area (red dots). Green light area represents the estimated microemulsion area. Increasing the water proportion (to the left side of the diagram) led to the creation of an emulsion system.

From our previous experience, different proportions of water were incorporated by titration in a system where the oily phase consisted of a commercially available CBD oil for topical use containing CBD at a dose of 20% *w*:*v*, with hemp oil as the main vehicle. As previously described, each mixture was titrated carefully under continuous stirring at a temperature of 30 °C to achieve equilibrium of liquid phases, taking care not to incorporate air bubbles into the formulation. The obtained preparation was then visually assessed for transparency and further titrated over the entire phase region. Figure 1 shows the pseudo-ternary phase diagram for all the mixtures described in Table 1 and the titration lines.

As can be seen from the resulting microemulsion area, many compositions were obtained that included high proportions of water or oily phases, which can be modulated in accordance with the requirements of the future formulation. Further titration with water led to the formation of an emulsion (white area on the left side of the diagram). The confirmation of the microemulsion area was based not only on the transparency and external appearance of the formulation, but also on the droplet-size distribution, which was determined by light scattering and conductivity results. 

**Figure 2:**
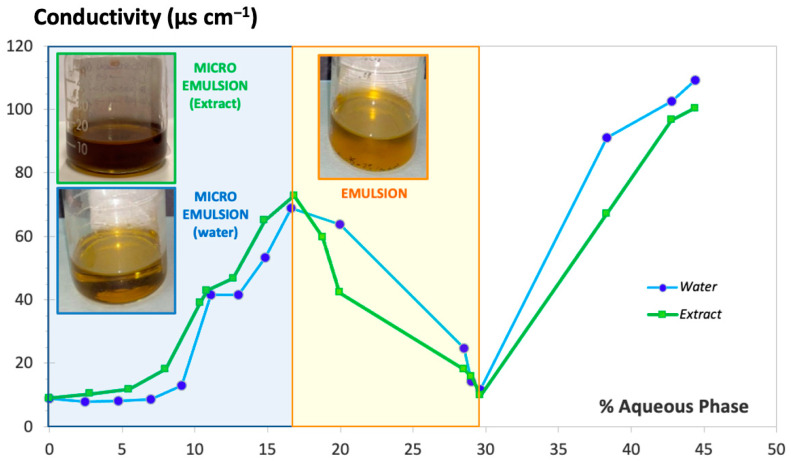
Conductivity value vs. hydrophilic phase proportion added under continuous stirring. Milli-Q^®^ water (blue line) and aqueous extract of *S. ebulus* (green line).

Figure 2 shows the conductivity values obtained (expressed in µS·cm^−1^) during titration with water over the oily phase mixed with S-CoS, starting with a composition S-CoS/O/W of 70/30/0% (*w*/*w*/*w*) and a total initial weight of 20 g (blue line). Identical measurement of conductivity was carried out with mixtures where the hydrophilic phase was substituted by the aqueous extract of *S. ebulus* previously described (green line).

The resulting conductivity-vs.-water or aqueous extract content plot showed a clearly different zone in both cases (light blue background on the left side of Figure 2) in which an increase of conductivity was obtained with up to 4 mL of hydrophilic phase added to the oily phase containing the surfactant/cosurfactant; the resultant formulation showed behavior clearly indicative of a microemulsion system. Conductivity values decreased dramatically when the volume of hydrophilic phase added during the titration ranged from 4 to 8.5 mL (light yellow background zone of the plot) as a result of the formation of a thicker and opaque system denoting the formation of an emulsion. Volumes of water greater than 8.5 mL (white zone of the plot) resulted in a new increase of conductivity associated with the breakage of the emulsion, which resulted in the formation of two separate phases. It was observed that the physical behaviors of the systems including water or the aqueous extract were practically identical.

**Figure 3:**
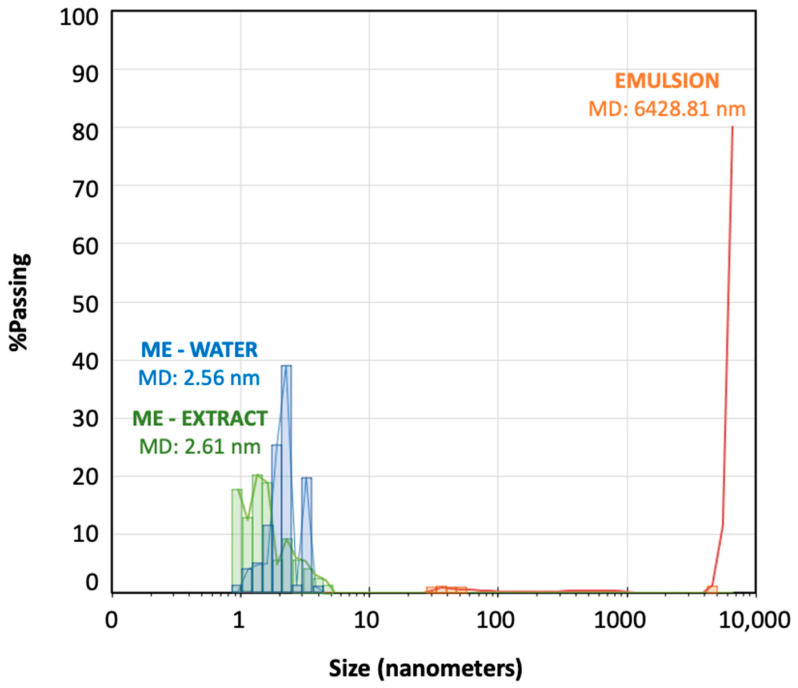
Droplet-size distribution (nanometers) for the microemulsion–extract (green bars), microemulsion–water (blue bars) and an emulsion (orange bars) obtained during the titration processes.

Droplet-size distribution measured by laser scattering was very useful as a method to confirm the microemulsion area of the pseudo-ternary phase diagram. Figure 3 shows the droplet-size distribution for the microemulsion–water (titration point S-CoS/O/W: 62/27/11% represented by blue bars) and for a subsequent emulsion obtained from the titration process (point S-CoS/O/W: 56/24/20%, represented by orange bars). The measurement was repeated under the same proportions for a microemulsion containing the vegetal aqueous extract as the hydrophilic phase (green bars). The mean diameter volume/surface area (Sauter diameter) was determined for each system.

As can be seen, both microemulsions prepared with water containing the *S. ebulus* extract showed droplets ranging from 0.9 to 6 nanometers with very similar mean diameter values. Increasing the water content led to an emulsion showing a minority group of droplets higher than 30 nm and a vast group of droplets higher than 5000 nm, what provided a mean diameter higher than 6000 nanometers. It was shown the formation of an emulsion when the proportion of aqueous phase was increased, but also, it could be demonstrated that the physical structures of both microemulsions (with water or with the hydrophilic extract was practically identical. These data revealed that the precipitation of any compounds from solution in the plant extract by migration to the oil phase was negligible. 

### 3.2. Rheological Properties

The rheological behavior of microemulsions has been widely studied. It has been described that the kind of structure conforming the system, whether bicontinuous or discontinuous, conditions the rheological characteristics of the microemulsion, as well as the interaction between the different aggregates. Generally, it is widely accepted that microemulsions exhibit Newtonian behavior (constant viscosity values) at low-to-medium shear rates but that this behavior is modified at high shear rates, especially for bicontinuous systems, due to a probable fragmentation of the structure, which leads to shear thinning or pseudoplastic behavior [69]. It has been also described that the proportion of the oil phase can modify the rheological behavior of microemulsions [70]. 

**Figure 4:**
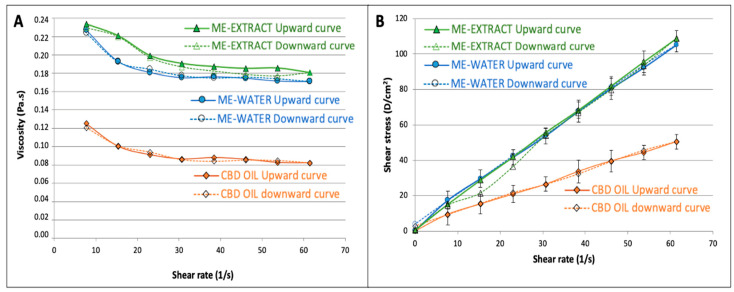
Viscosity-vs.-shear rate profiles showing the mean viscosity value (**A**), and shear stress-vs.-shear rate upward and downward rheograms (**B**).

Our data revealed Newtonian behavior for the CBD oil, as well as for our selected microemulsions (61:27:11). SS-vs.-SR plots show good linearity (see Figure 4B), and a constant viscosity could be calculated for both systems. The average value of viscosity obtained at shear rates ranging from 23 to 61 s^−1^ was used as a reference viscosity because it was observed that this value did not differ significantly from the viscosity obtained from the linear regression of SS-vs.-SR rheograms. The results from the rheological measurements were mathematically adjusted to the power-law model of Ostwald–de Waele (Equation (1)) using logarithmic plots, and the resulting coefficients for the CBD oil and for the microemulsions are shown in Table 2.

Although all formulations were very fluid, showing good spreadability, it can be observed that viscosity value for the microemulsion-water was approximately double that obtained for the CBD oil, probably due to the presence of a high proportion of surfactant/cosurfactant. Apparent viscosity was slightly higher for the microemulsion extract in comparison to that of the formulation with water due to the higher consistency of the extract in the aqueous phase. Nevertheless, the consistency coefficient did not show statistically significant differences between the CBD oil and the two microemulsions studied due to the high variability found. 

Comparing the flow-behavior indices, it could be observed that all exhibited near-Newtonian behavior. Nevertheless, it was found that the n values obtained for both microemulsions were statistically lower than 1, which denotes slight pseudoplastic behavior. The viscosity vs. shear rate diagrams (Figure 4A) showed a transition from Newtonian to non-Newtonian fluid behavior: both microemulsions behaved as a shear-thinning fluids at higher shear rates, a finding that agrees with the behavior found by other authors studying microemulsions [70]. After adjusting the Herschel–Bulkeley model to our data, it was found that the yield stress value did not differ from zero (*p* = 0.09) for both microemulsions. 

The area under the curve of the upward and downward curves, corresponding to the viscosity vs. shear rate to define the hysteresis, was used to estimate the thixotropy of the three formulations. It was found that the microemulsions showed significantly higher (3 times higher hysteresis, see Table 2) thixotropic behavior, which indicates that the microemulsions require more energy to reorganize their structures when they are exposed to higher shear rates. The oil does not have such a complicated structure, as is shown from the differences found in this parameter. Nevertheless, the thixotropy is also very low in the microemulsions, which show high recuperative ability at the studied shear rates.

In order to study the physical stability of the microemulsion including the *S. ebulus* extract as the aqueous phase, its rheological characteristics were measured at 12 months after the formulation had been stored under standard conditions of darkness and a temperature of 25.0 ± 0.5 °C. Mean apparent viscosity was 0.188 ± 0.001 Pa·s. No statistically significant differences were found for this parameter and for the other determined rheological values (n = 0.93 ± 0.11, hysteresis = 0.251 ± 0.135 Pa). These data confirmed that no structural changes took place in the microemulsion system during the storage period, which confirmed the good stability of the formulation. No signs of separation of phases or precipitation were detected.

### 3.3. Compatibility Studies by DSC

**Figure 5:**
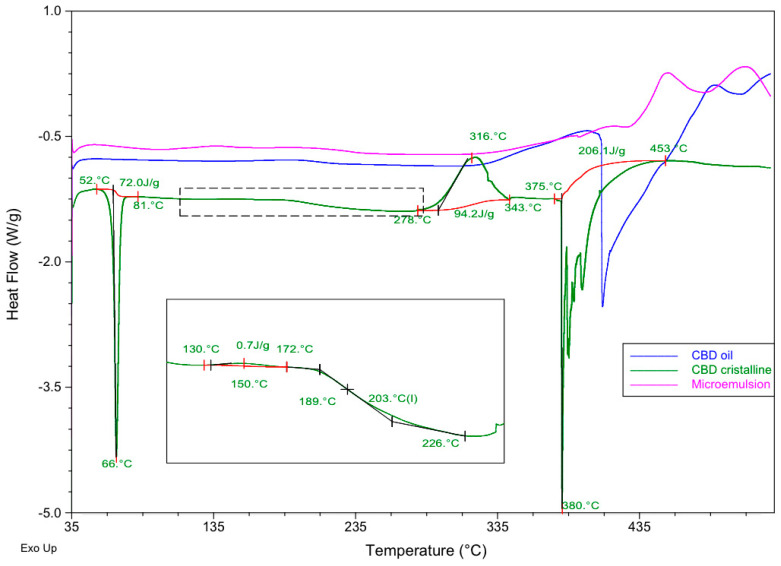
Differential scanning calorimetry (DSC) curves of pure CBD crystalline (green line), CBD oil (blue line) and the microemulsion containing the same commercial oil as the oily phase (pink line). Red curve denotes base lines.

DSC experiments were conducted as previously described for three samples: pure CBD in crystalline original form, a commercial CBD oil, and a microemulsion containing the CBD oil as the oily phase, a surfactant-cosurfactant 1:1 mixture and water at proportions of (27:31:31:11) (*w*:*w*:*w*:*w*). Thermograms were obtained from 35 to 535 °C at a heating rate of 10 °C/min to ensure that all possible melting or decomposition processes could be analyzed. Table 3 and Figure 5 describe the main thermal processes found.

As can be seen, CBD crystals presented an endothermic process that corresponds to melting at 66 °C within a range of temperatures of 52–81 °C (T_onset_ and T_enset_) and an enthalpy value of 72.0 J/g. The DSC profile showed an endothermic event, indicating the decomposition of the DSC at a peak temperature of 380 °C ranging from 375 to 453 °C (T_onset_ and T_enset_) and an ∆H = 206.1 J/g. A slight exothermic event was also found at 150 °C, and a significant exothermic peak was observed at 316 °C. A possible glass transition was observed from 189 to 226 °C. These details introduce some differences in comparison to the DSC profile obtained for CBD by other authors [71].

The DSC line corresponding to the commercial oil containing 20% CBD in solution (blue line in Figure 5) showed a slight exothermic process from 126 to 234 °C (T_peak_ = 192 °C) and a majoritarian endothermic event indicating the decomposition of the components at 409 °C within a range of temperatures from 398 to 489 °C.

The microemulsion profile showed different behavior, with a slight endothermic process at 393 °C with ∆H = 1.8 J/g, a majoritarian exothermic event at 451 °C ranging from 427 to 480 °C (T_onset_ and T_enset_) and a resulting enthalpy of 55.5 J/g. This thermal profile showed that the microemulsion was more stable than the commercial oil, both having the CBD in solution.

In the light of these findings, no incompatibilities were detected between CBD and the components of the CBD oil and the microemulsion. Although these results are promising at this point of a pre-formulation study, they must be considered cautiously. Further techniques like isothermal stress testing–Fourier transform infrared spectroscopy or quantitative analysis after storage under stressed conditions [63,72] should be used to examine the compatibility of the drug once a complete formulation is designed.

### 3.4. In Vitro Drug Release

The in vitro release studies were performed to characterize the release of CBD from the commercially available CBD oil and to elucidate whether or not the creation of a microemulsion system could modify significantly the retention or release of the CBD in comparison to its behavior in the original CBD oil. The release of CBD from the oil phase was studied concomitantly with the release of CAF from the aqueous phase, as CAF was used as a model hydrophilic antioxidant compound found in the *S. ebulus* extract and here used in the microemulsion.

Apparent steady-state intrinsic flux (J_app_ in µg/cm^2^·h) was calculated for each experiment by non-linear regression using a bi-exponential model from the flux calculated at each time interval from Equation (3) and following the mathematical approach proposed by many authors for the evolution of the intrinsic flux over experimental time and in flow-through diffusion cells [73,74]. The permeation coefficient (Kp in cm·h^−1^) was calculated from the J_app_ value divided by the donor concentration at time zero, according to the Fickian diffusional model.

In order to calculate not only the global release process from the Kp value, but also the release of CBD from the formulation, the parameter K1, which represents the release kinetic from the formulation expressed in h^−1^, was determined by non-linear curve fitting of the cumulative amounts permeated vs. time profile according to Equation (4), which was derived from the first-order consecutive reactions mathematical approach [66]. The SIMFIT statistical package was used for the nonlinear curve fitting by weighed least squares, selecting the model described as a monomolecular growth-GCFIT model. Information on goodness of fit was also provided by the software to determine the reliability of the constants obtained.

A previous experiment was carried out using a CBD solution with a concentration of 1 mg·mL^−1^ in an ethanol:water 70:30 (*v*:*v*) mixture, and the same medium was used in the receptor chamber to ensure that the solubility is exactly the same on both sides of the diffusion membrane. This experiment permitted us to characterize the diffusional parameters of the previously dissolved CBD without the interference of a formulation that could condition its release from its structure or modify the solubility and hence the diffusion process. In our study, the flow rate of the receptor medium (F_rec_ of Equation (3)) was 1 mL·min^−1^ and the temperature of the cell warmer was fixed to 37 °C. The receptor fluid was collected every hour for the first 10 h and then every 4 h up to 24 h. The amount of CBD that had permeated at each time point was analyzed by the previously described HPLC method. All experiments were conducted in sextuplicate.

Once the diffusional properties of CBD in solution were determined, in vitro release experiments were carried out for the commercial CBD oil and for the microemulsions under the same conditions described for the previous experience with the CBD solution.

Table 4 describes the main diffusional and release parameters for CBD dissolved in the commercial oil and for CBD in the microemulsion with water. The same table also includes the values related to the microemulsion containing the *S. ebulus* extract as the aqueous phase, simultaneously analyzing the release parameters of CBD from the oil phase and those of CAF from the extract. 

**Figure 6:**
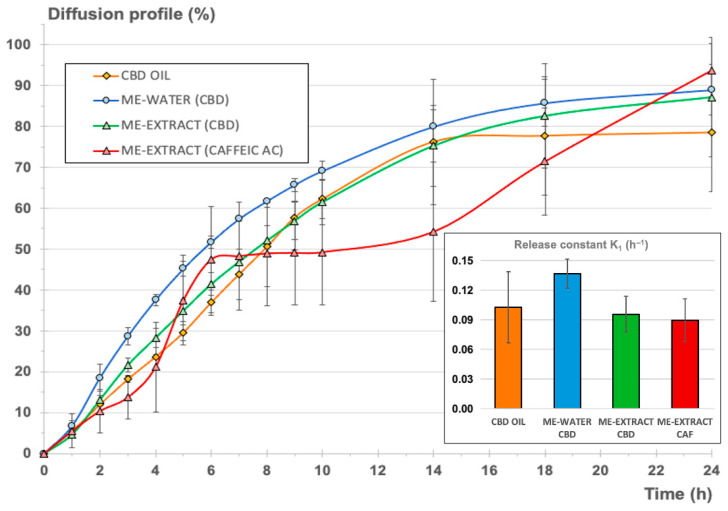
Cumulative diffusion profile as a percentage for the three formulations (CBD oil—orange, microemulsion with water—blue, microemulsion with the extract—green). The cumulative diffusion profile of CAF from the microemulsion with the extract was also included for comparison (red). Right-side bar chart: release constant (h^−1^) ± confidence interval (*p* < 0.05).

Figure 6 shows the comparative release profiles for the two systems analyzed, which are expressed as cumulative amount permeated vs. time in percentage of the total amount of CBD added to the donor chamber for each experiment for normalization. For the second microemulsion including the vegetal extract, the release profiles of CBD and CAF were determined simultaneously.

As can be seen, steady-state intrinsic flux values were very different in each case, as expected, considering the different initial CBD concentrations in the donor chamber. Nevertheless, permeability coefficients for CBD from the oil and the microemulsions were very similar, suggesting that the full CBD release and diffusion processes were similar for the oil and the microemulsions. It was also observed that the estimated time necessary to reach the steady state was almost double for the microemulsions in comparison to the value calculated for the oil. These results indicates that the microemulsion system does not create a barrier to the release of CBD but provides a slight delay before attainment of the steady state, where the maximum apparent intrinsic flux is reached.

Comparing the permeability coefficient Kp for CBD and CAF from the emulsion containing the extract, it can be observed that global diffusion was one order of magnitude greater (more than 20 times greater) for CBD.

Table 4 also shows the main release parameters calculated from the cumulative amounts permeated vs. time profile, adjusting the monomolecular growth-fitting exponential model to the experimental data. Goodness of fit was good enough in all cases. The Durbin–Watson statistic yielded values below 2.5, denoting good correlation except for the microemulsion with water, for which the value obtained was a bit higher. Nevertheless, the rest of the fitting parameters confirmed the good quality of the fit. Shapiro–Wilks values were greater than 0.05, confirming the normality of the data. It could be concluded that the calculated constants from the model were consistent and reliable in all cases. Higher variability was observed for the release model calculated for CAF, but the goodness of the curve-fitting was also acceptable.

The normalized diffusion profile, expressed as cumulative percentage of each compound (CBD or CAF) diffused with respect to the initial amount in the donor chamber (see Figure 6), reveals that the inclusion of the CBD oil in a microemulsion with a hydrophilic phase (blue and green lines) resulted in a profile similar to the normalized profile corresponding to the original CBD oil (orange profile). To refine this result, K_1_ release constants were determined from the cumulative amount of CBD diffused vs. time profiles according to Equation (4), assuming first-order consecutive kinetics for the comparison of all formulations. This kinetic can be assumed, considering that diffusion through a membrane starting from a drug in solution is taking place. Besides, the release of a certain drug from a microemulsion is likely to adopt pseudo-zero-order kinetics, but these kinetics will be conditioned to the volume of the dispersed phase, the partitioning of the drug between the interphases, and the diffusion rate of the drug [75]. 

Comparing the calculated K_1_ values for CBD release from the oil and the microemulsions, it could be concluded that no statistically significant differences were found. The inclusion of the oil in a stable structure with a high proportion of surfactant/cosurfactant mixture and a aqueous phase did not result in significant modification of the release of the CBD, with the advantage that hydrophilic compounds can be included in this stable system without provoking significant retention of CBD or other non-desirable phenomena like the insolubilization of the hydrophilic or lipophilic compounds or the breakdown of the structure, as can be seen in Figure 6, which shows the results of the microemulsion produced with the aqueous vegetal extract.

Comparing the resulting release parameters for CBD and CAF, it could be observed that the release constants (K_1_) were very similar and that no statistically significant differences were found (*p =* 0.05). A pseudo-plateau was observed in the mean data between 6 and 14 h (red profile in Figure 6), but this observation was considered to be negligible due to the higher variability observed on the CAF profiles. It can be concluded that the microemulsion, in comparison to the oil, did not create a barrier to the release of CBD and CAF, as both showed similar release-rate values, also considering the different nature of both compounds and the different initial doses in the microemulsion. Subsequent ex vivo and in vivo pharmacokinetic studies are necessary to characterize the final formulation.

### 3.5. Antioxidant Activity and Total Phenolics of the Microemulsion-Extract

Total phenolics content (TPC) and total antioxidant capacity (TAC) of the microemulsion containing the *S. ebulus* extract as the aqueous phase were determined, as described, to be under 2.5. Both studies were conducted at time zero from the just-prepared microemulsion and from the microemulsion stored for 12 months under room-temperature storage conditions in sealed containers in the absence of light to elucidate whether or not the antioxidant activity of the extract was preserved when it was formulated in the microemulsion. Table 5 shows the resulting TPC and TAC mean values for each situation.

No statistically significant variability was found in the total phenolics content and the total antioxidant capacity after 12 months, which confirmed that the integrity of the antioxidant compounds after extraction with methanol and the subsequent inclusion of the extract into a microemulsion system was preserved under the previously described storage conditions. As previously mentioned, no insolubilization phenomena were observed after up to 12 months under standard storage conditions and no breakdown of the structure turbidity was observed.

## 4. Conclusions

It is widely described that CBD and any other cannabinoids can play an important role in the treatment of different skin disorders due to the importance of the endocannabinoid system in the skin [1,32,76]. The lipophilicity of CBD makes it very easy to formulate an oil or a semisolid oily preparation for topical use, but no hydrophilic compounds could be included. The formulation of an emulsion could be an option for the creation of a cream, but there exist inherent advantages of microemulsions, which are easier to formulate at industrial scale, are thermodynamically stable and exhibit lower viscosity and Newtonian flow, while emulsions are thermodynamically unstable and show higher viscosity [77].

In our study, microemulsions made from commercial CBD oil have been successfully developed and characterized in terms of a pseudo-ternary phase diagram to find the correct microemulsion composition based on additional confirmatory data of conductivity, droplet size and rheological characteristics. It was also found that the viscosity of the designed microemulsion was suitable for topical administration, showing slight pseudoplastic behavior. The inclusion of a vegetal extract as the aqueous phase did not significantly modify the rheological properties in comparison to those of the original CBD oil, maintaining a CBD load bigger than 5% *w*:*w*. The physical integrity and stability of the microemulsion system was also shown, as the rheological parameters were not significantly altered after 12 months’ storage.

Many strategies for the vehiculization of CBD based on nanoemulsions (thermodynamically unstable), and other lipid-based formulations described in an exhaustive and very interesting recently published review [78] included only one microemulsion structured in a gel and loaded with 1% CBD. The microemulsions developed in the present study provide a wide variety of proportions, with a significantly higher load of CBD. As can be seen, the formulation used as a reference for physicochemical characterization in our study contained more than 5% of CBD, and other compositions contained up to 8.4% CBD. It must be also taken into consideration that there exists the possibility of working with a CBD oil containing 30% CBD instead of 20%, which could give us the option of producing a microemulsion with a final load of CBD greater than 12% for the last composition described in Table 1. This versatility and load ability can result in a increase in transdermal penetration in comparison to other reported formulations.

Also studied were the release and retention of CBD from the microemulsions in comparison to the original oil. The creation of the bi-continuous system did not significantly affect the release of the CBD. The release of caffeic acid as a model hydrophilic compound was found to be similar to that observed for CBD. It can be concluded that it was possible for the developed microemulsion to include CBD in combination with different hydro-soluble compounds with potential antioxidant and anti-inflammatory effects without creating a barrier that could impede their release from the topical formulation.

It was demonstrated that the microemulsion containing the vegetal extract of *S. ebulus* ripe fruits showed good antioxidant capacity that was preserved after 12 months’ storage in terms of total phenolic compounds and total antioxidant capacity.

This strategy appears as a promising galenical alternative due to its inherent advantages as a drug carrier for both hydrophilic and lipophilic substances, with high stability and simplicity compared to other kinds of formulations. The microemulsion here designed and characterized has unique potential pharmacological properties for future therapeutic uses.

## Figures and Tables

**Table 1:** Composition of the start point and of the limit of microemulsion area for each titration line used for the preparation of the pseudo-ternary diagram, including the resulting CBD content for each composition. A higher water proportion led to the formation of an emulsion.

Starting Composition S-CoS (1:1)/Oily Phase (% *w*/*w*)	Limit of Microemulsion Area S-CoS/O/W (% *w*/*w*)	CBD Content (% *w*/*w*)
90/10	51/6/43	1.2
80/20	62/16/22	3.2
75/25	66/22/12	4.4
70/30	62/27/11	5.4
65/35	60/32/8	6.4
60/40	57/38/5	7.6
55/45	51/42/7	8.4

**Table 2:** Rheological properties of the original CBD oil in comparison to the data obtained for the microemulsion systems.

Parameters	CBD OIL	Microemulsion–Water	Microemulsion–Extract
Mean apparent viscosity, (Pa·s)	0.086 ± 0.004 ^a^	0.176 ± 0.004 ^b^	0.186 ± 0.002 ^c^
Consistency coefficient, K (Pa·s^n^)	2.02 ± 1.70 ^a^	2.92 ± 1.31 ^a^	2.81 ± 0.20 ^a^
Flow-behavior index, n	0.86 ± 0.21 ^a^	0.88 ± 0.11 ^a^	0.94 ± 0.04 ^a^
Determination coefficient R^2^ (%)	99.07	99.64	99.92
Hysteresis (Pa)	0.072 ± 0.043 ^a^	0.223 ± 0.078 ^b^	0.313 ± 0.116 ^b^

^a/b/c^ Different letters in the same line denote a statistically significant difference (*p* < 0.005). Values are expressed as mean ± confidence interval 95% (*n* ≥ 3).

**Table 3:** Thermo-analytical DSC data for CBD crystals, the commercial CBD 20% oil and microemulsion–water.

Sample ID	Reaction	T_onset_ (°C)	T_endset_ (°C)	T_peak_ (°C)	Enthalpy (J/g)
CBD crystalline	Endothermic	52	81	66	72.0
	Exothermic	130	172	150	0.7
	Tg	189	226	203	-
	Exothermic	278	343	316	94.2
	Endothermic	375	453	380	206.1
CBD oil 20%	Exothermic	126	234	191	10.1
	Endothermic	398	489	409	415.6
Microemulsion	Endothermic	390	411	393	1.8
	Exothermic	427	480	451	55.5

**Table 4:** In vitro diffusion and release parameters for each formulation.

Parameter	20% CBD Oil	Microemulsion–Water (CBD)	Microemulsion–Extract (CBD)	Microemulsion–Extract (Caffeic Acid)
SS-Intrinsic Flux—J_app_ (µg/cm^2^·h) ^1^	1604.403	354.057	306.122	177.583
Time to reach SS (T_Jmax_) (h)	0.648	1.263	1.468	1.680
Permeability coefficient Kp (cm/h)	0.0054	0.0079	0.0057	0.0002
**Cumulative amount vs. time profile curve fitting (SIMFIT monomolecular growth):**				
Asymptote (mg)	13.998 ± 1.024	2.112 ± 0.043	2.699 ± 0.112	0.115 ± 0.014
Release constant K1 (h^−1^)	0.103 ± 0.036	0.137 ± 0.007	0.096 ± 0.008	0.0896 ± 0.022
Goodness of fit: R-squared	0.9219	0.9921	0.9518	0.7324
Durbin-Watson test statistic	2.2946	2.669	1.4788	1.0568
Shapiro-Wilks W	0.965	0.974	0.909	0.952

^1^ Apparent steady-state intrinsic flux.

**Table 5:** Antioxidant activity of the *S. ebulus* extract in the microemulsion system at 0 and 12 months of storage time, expressed as TPC and TAC.

Antioxidant Activity (µg eq. GA/µg Dry Extract)	Storage Time	
	t = 0	t = 12 Months
TPC (Folin-Ciocalteau) µg eq. GA/µg dry extract	0.0742 ± 0.0009 ^a^	0.0712 ± 0.0006 ^b^
TAC (CUPRAC) µg eq. GA/µg dry extract	0.0407 ± 0.0055 ^a^	0.0437 ± 0.0135 ^a^

^a/b^ Different letters in the same line denotes a statistically significant difference (*p* < 0.05). Values are expressed as mean ± Confidence interval 95% (*n* = 6).

## Data Availability

Data are contained within the article. The data presented in this study are also available on request from the corresponding author.
